# Integrative genomic analysis of N6-methyladenosine-single nucleotide polymorphisms (m^6^A-SNPs) associated with breast cancer

**DOI:** 10.1080/21655979.2021.1935406

**Published:** 2021-06-21

**Authors:** Zixue Xuan, Yiwen Zhang, Jinying Jiang, Xiaowei Zheng, Xiaoping Hu, Xiuli Yang, Yanfei Shao, Guobing Zhang, Ping Huang

**Affiliations:** a Department of Pharmacy, Zhejiang Provincial People’s Hospital, People’s Hospital of Hangzhou Medical College, Hangzhou, China; b Key Laboratory of Endocrine Gland Diseases of Zhejiang Province, Zhejiang Provincial People’s Hospital, People’s Hospital of Hangzhou Medical College, Hangzhou, China

**Keywords:** m^6^A, single nucleotide polymorphisms (SNPs), breast cancer, GWAS, m^6^Avar

## Abstract

Due to the important role of N6-methyladenosine (m^6^A) in breast cancer, single nucleotide polymorphisms (SNPs) in genes with m^6^A modification may also be involved in breast cancer pathogenesis. In this study, we used a public genome-wide association study dataset to identify m^6^A-SNPs associated with breast cancer and to further explore their potential functions. We found 113 m^6^A-SNPs associated with breast cancer that reached the genome-wide suggestive threshold (5.0E-05), and 86 m^6^A-SNPs had eQTL signals. Only six genes were differentially expressed between controls and breast cancer cases in GEO datasets (GSE15852, GSE115144, and GSE109169), and the SNPs rs4829 and rs9610915 were located next to the m^6^A modification sites in the 3ʹUTRs of *TOM1L1* and *MAFF*, respectively. In addition, we found that polyadenylate-binding protein cytoplasmic 1 might have a potential interaction with rs4829 (*TOM1L1*) and rs9610915 (*MAFF*). In summary, these findings indicated that the SNPs rs4829 and rs9610915 are potentially associated with breast cancer because they had eQTL signals, altered gene expression, and were located next to the m^6^A modification sites in the 3ʹUTRs of their coding genes. However, further studies are still needed to clarify how genetic variation affects the epigenetic modification, m^6^A, and its subsequent functions in the pathogenesis of breast cancer.

## 1 Introduction

Breast cancer is the most frequently diagnosed cancer and the leading cause of cancer-related death among women worldwide [1,2]. Numerous potential risk loci and susceptible genes have been identified by genome-wide association studies (GWASs) in breast cancer [3,4]. Presently, high-penetrance genes (*BRCA1, BRCA2, CDH1, STK11, PTEN*, and *TP53*) and moderate-penetrance genes (*ATM, CHEK2, PALB2*, and *BRIP1*) are known to genetically predispose individuals to the development of breast cancer [5]. Recently, a growing number of studies have suggested that genetic variations, especially single nucleotide polymorphisms (SNPs), contribute to breast cancer susceptibility [6,7]because SNPs located in protein-coding regions, as well as multiple SNPs located in non-coding regions, can alter gene expression and participate in epigenetic regulation.

N6-methyladenosine (m^6^A) modification is the most abundant internal modification of messenger RNA (mRNA) and non-coding RNA, such as microRNA, long non-coding RNA, and circular RNA in eukaryotes [8,9]. Emerging evidence has shown that m^6^A is involved in RNA metabolism and is dynamically regulated by methyltransferases, demethylases, and binding proteins [10,11]. Furthermore, it was confirmed that m^6^A plays an important role in breast cancer [12,13]. For example, the m^6^A demethylase FTO promoted breast cancer progression by inhibiting BNIP3^13^, and the HBXIP-elevated methyltransferase METTL3 promoted breast cancer progression by inhibiting the tumor suppressor let-7 g [12]. Recent studies have also suggested that SNPs may affect m^6^A by altering the RNA sequences of the target sites or crucial nucleotides [14] and also contribute to the progression of breast cancer. Importantly, m^6^A-SNPs have the potential to regulate gene expression and mRNA stability, consequently leading to disease [15,16]. Yang et al. found that rs2416282 contributed to esophageal squamous cell carcinoma (ESCC) risk by regulating YTHDC2 expression [16]. Recently, another study found that the SNP rs5746136 affected m^6^A modification and influenced the expression of SOD2 in bladder cancer [15], and some *METTL14* SNPs were similarly related to neuroblastoma [17]. Zhuo also reported that *WTAP* SNPs may be genetic modifiers in hepatoblastoma [18]. Ren et al. reported that the rs8400 AA genotype was correlated with increased expression of *ALKBH5* and may be a risk factor for hepatoblastoma in the clinical stage III + IV subgroup [19]. In glioma, studies found the m^6^A-SNPs, rs7766006 (*WTAP*), rs3738067 (*YTHDF2*), and rs9939609 (*FTO*), to increase cancer risk, whereas rs2293595 (*YTHDC1*), rs3813832 (*YTHDC1*), and rs8047395 (*FTO*) reduced the risk [20].

Given that m^6^A-SNPs play a pivotal role in cancer [18,21–23] and the effect of m^6^A -SNPs in breast cancer is unclear, identifying m^6^A -SNPs associated with breast cancer is necessary and could provide a new annotation for the pathogenic mechanism of breast cancer risk loci identified by GWAS. Therefore, this study aimed to identify m^6^A-SNPs associated with breast cancer using a public GWAS database and the m^6^AVar database, and to demonstrate their potential functions.

## 2 Materials and methods

### 2.1 Screening for m^6^A -SNPs associated with breast cancer

The m^6^AVar database provides information for the annotation, visualization, and exploration of variants associated with m^6^A [24,25]. In the m^6^AVar database, variants were defined at three confidence levels, namely high confidence level when RNA modification sites were directly obtained from the m^6^A-Label-seq, DART-seq, m^6^A-REF-seq, or miCLIP experiment; medium confidence level when RNA modification sites were obtained from the MeRIP-Seq or m^6^A-Seal-seq experiment; and low confidence level when RNA modification sites were obtained by prediction of the whole transcriptome.

The intersection of SNPs, which reached the genome-wide suggestive threshold (5.0E-05), between the breast cancer GWAS and m^6^Avar databases was used to explore m^6^A-SNPs associated with breast cancer. In this study, breast cancer GWAS data were downloaded from the website (ftp://ftp.ebi.ac.uk/pub/databases/gwas/summary_statistics/MichailidouK_29059683_GCST004988/), and the m^6^A-SNP list was obtained from the m^6^Avar database (http://rmvar.renlab.org/download.html).

### 2.2 Expression quantitative trait loci (eQTL) analysis of m^6^A-SNPs

eQTL analysis can be used to evaluate the potential function of m^6^A-SNPs with eQTL signals in transcription regulation, such as altering protein binding, changing motifs, and affecting deoxyribonuclease. Hence, eQTL analysis was performed to investigate the breast cancer-associated m^6^A-SNPs that could affect RNA modification using the HaploReg browser (https://www.encodeproject.org/software/haploreg/) [26,27].

### 2.3 Gene Ontology (GO) enrichment analysis

GO enrichment analysis was performed using the Metascape tools (https://metascape.org/gp/index.html#/main/step1). GO biological process, GO molecular function, and GO cellular component were used to classify these genes according their functional annotation [28].

### 2.4 Differential gene expression analysis

The GSE15852, GSE115144, and GSE109169 datasets were downloaded from NCBI GEO (https://www.ncbi.nlm.nih.gov/geo/) [29,30]. GEO2R (https://www.ncbi.nlm.nih.gov/geo/geo2r/) was used to identify the differentially expressed genes corresponding to m^6^A-SNPs with eQTL between controls and breast cancer cases. A significance level of 0.05 and |LogFC|≥2 were used for differential expression analysis.

### 2.5 Prediction of m^6^A-SNPs affecting RNA modification

To determine whether the m^6^A-SNPs affected RNA modification and predicted changes in m^6^A modification, we used the sequence-based RNA adenosine methylation site predictor (SRAMP), which is an online m^6^A modification prediction tool [31]. Further, the USCS browser (GRCh37/hg19; https://genome.ucsc.edu/) includes abundant assemblies and annotations of the genomes of vertebrates and model organisms. Therefore, we used it to analyze the potential functions of m^6^A-SNPs [32,33].

## 3 Results

### 3.1 Identification of m^6^A-SNPs associated with breast cancer

**Figure 1.**
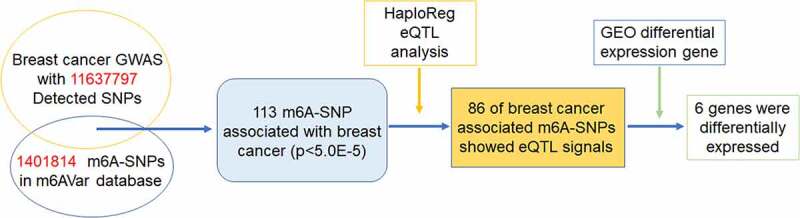
Flow chart of study design and analysis

**Figure 2.**
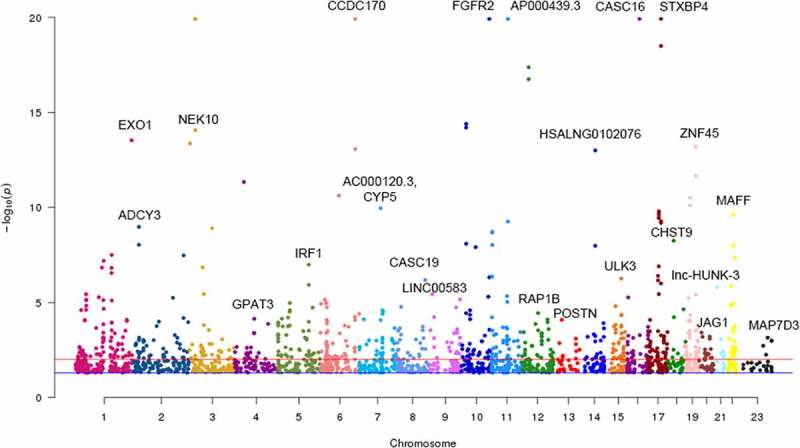
Manhattan plot of identified m^6^A-SNPs associated with breast cancer. The Manhattan plot showed – log10(p.value) for each of 17,599 m^6^A-SNPs associated with breast cancer. There were 285 m^6^A-SNPs associated with breast cancer (p < 0.001), and 113 m^6^A-SNPs associated with breast cancer (p < 5.0E-5)

To identify m^6^A-SNPs associated with breast cancer, the intersection of the GWAS and m^6^AVar databases was analyzed. Of the 11,637,797 SNPs and 1,401,814 m^6^A-SNPs in the breast cancer GWAS and m^6^A Var databases (Figures 1 and 2), we identified 113 m^6^A-SNPs, which reached the genome-wide suggestive threshold (5.0E-05), to be associated with breast cancer (Table S1).

To check whether these m^6^A-SNPs have eQTL effects, the HaploReg browser was used, finding that 86 of these breast cancer-associated m^6^A-SNPs had eQTL signals. Subsequently, we found that of the 86 m^6^A-SNPs with eQTL signals, 31 belonged to the high confidence category, 20 to the medium confidence category, and 35 to the low confidence category. Additionally, among these 86 m^6^A-SNPs, 72 were loss-of-function SNPs and 14 were gain-of-function SNPs (Table S1).

**Figure 3.**
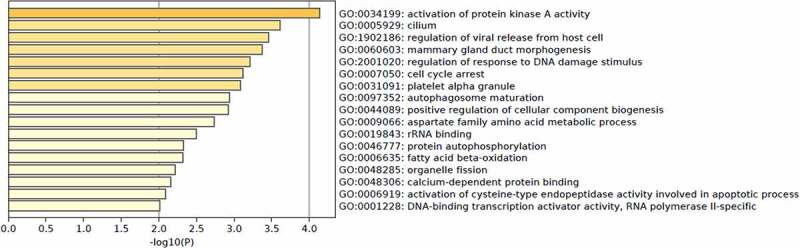
GO enrichment analysis was performed to annotate the potential function of genes corresponding to identified m^6^A-SNPs

According to GO enrichment analysis, we found that the molecular functions of these m^6^A-SNPs were associated with rRNA binding, calcium-dependent protein binding, DNA-binding transcription activator activity, and RNA polymerase II-specific binding. In addition, the functions of the genes with these m^6^A-SNPs were enriched in various biological processes, such as the activation of protein kinase A activity, mammary gland duct morphogenesis, and autophagosome maturation (Figure 3).

### 3.2 Six corresponding genes of eight m^6^A-SNPs were differentially expressed in breast cancer

**Figure 4.**
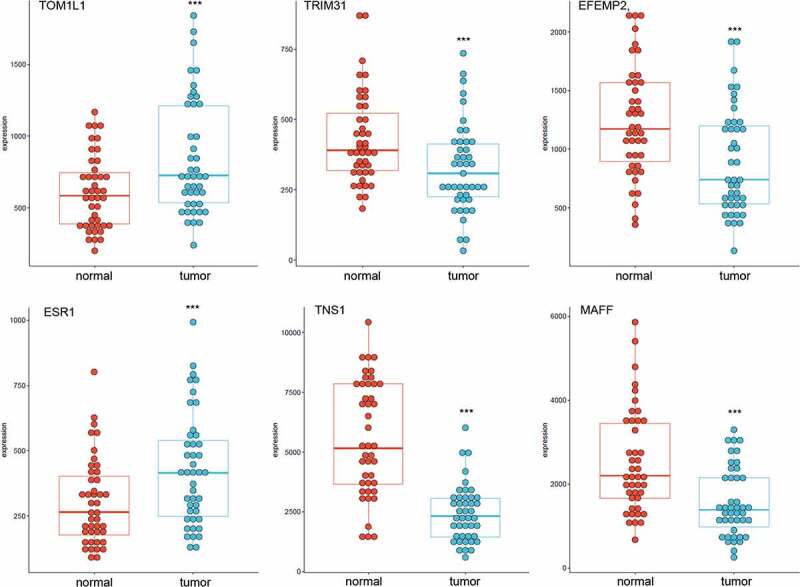
Expression levels of selected genes in GSE15852 dataset. *TOM1L1* and *ESR1* were highly expressed, but *MAFF, TNS1, EFEMP2*, and *TRIM31* were significantly downregulated in breast cancer

**Figure 5.**
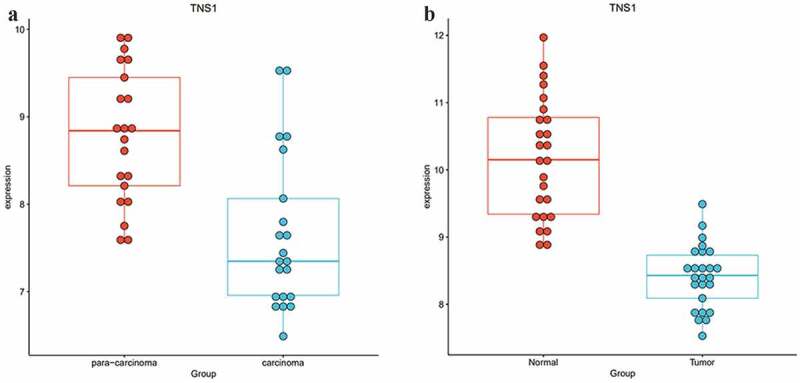
Expression levels of selected genes in GSE115144 and GSE109169 dataset, and we found only *TNS1* was differentially expressed in GSE115144 (a) and GSE109169 (b) datasets

To study whether these m^6^A-SNPs may affect the expression level of their corresponding genes, differential gene expression analysis using GEO2R was performed, finding that only six genes, namely *TOM1L1* (rs1802212; rs4829), *ESR1* (rs2747655), *MAFF* (rs2267372; rs9610915), *TNS1* (rs1424916), *EFEMP2* (rs2234458), and *TRIM31* (rs2023472), were differentially expressed in the GSE15852 dataset (p < 0.05) (Table 1). Specifically, *TOM1L1* and *ESR1* were highly expressed, but *MAFF, TNS1, EFEMP2*, and *TRIM31* were significantly downregulated in breast cancer (Figure 4). Moreover, we found that only TNS1 was differentially expressed in the GSE115144 and GSE109169 datasets (Figure 5). Taken together, these results suggest that eight m^6^A-SNPs (rs1802212, rs4829, rs2747655, rs2267372, rs9610915, rs1424916, rs2234458, and rs2023472) may affect the expression level of their corresponding genes.

**Table 1.** Six genes of eight m^6^A-SNPs associated with breast cancer were differentially expressed between controls and breast cancer in the GSE15852

SNP ID	Chr	Position	m6A_ID	Gene	Gene_ region	eQTL Hit	Confidence_level	Modification_ function	Pvalue
rs1802212	17	54961293	RMVar_ID_782932	TOM1L1	3’UTR	32	m6A-Seal-seq:(Medium)	Functional Loss	3.20E-19
rs2747655	6	152126186	RMVar_ID_1369593	ESR1	3’UTR	3	Prediction:(Low)	Functional Loss	8.62E-14
rs2267372	22	38202227	RMVar_ID_520365	MAFF	exon	35	MeRIP-seq:(Medium)	Functional Loss	2.49E-10
rs9610915	22	38215073	RMVar_ID_778207	MAFF	3’UTR	9	MeRIP-seq:(Medium)	Functional Loss	1.05E-08
rs1424916	2	217874577	RMVar_ID_814993	TNS1	intron	4	m6A-Label-seq:(High)	Functional Loss	3.36E-08
rs4829	17	54961954	RMVar_ID_781699	TOM1L1	3’UTR	14	miCLIP:(High)	Functional Loss	1.00E-06
rs2234458	11	65871903	RMVar_ID_919384	EFEMP2	intron	5	Prediction:(Low)	Functional Loss	4.68E-06
rs2023472	6	30108087	RMVar_ID_1375857	TRIM31	CDS	1	Prediction:(Low)	Functional Gain	1.83E-05

Abbreviations: m^6^A, N6-methyladenosine; UTR, untranslated region; Chr, chromosome; eQTL, expression quantitative trait loci.

### 3.3 TOM1L1 *(rs4829) and* MAFF *(rs9610915) may affect m^6^A modification in breast cancer*

**Figure 6.**
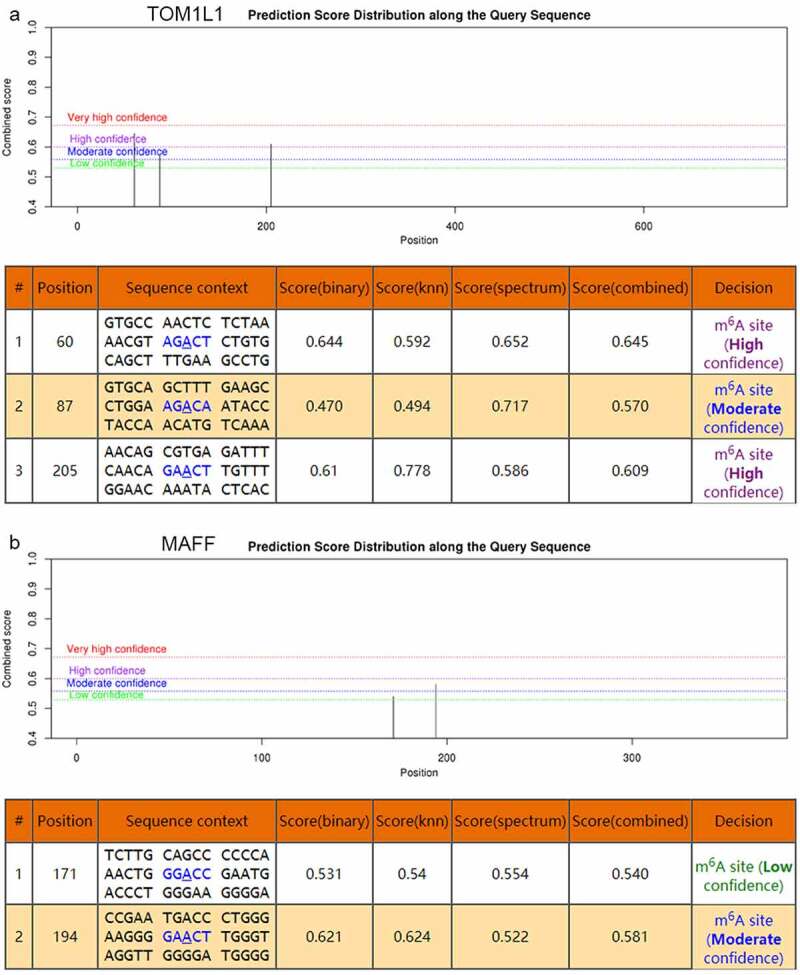
The genomic sequence of TOM1L1 and MAFF transcripts were used to predict the m^6^A modification on website (http://www.cuilab.cn/sramp). There were moderately-highly convincible m^6^A-modified predicted peaks near *TOM1L1* (rs4829) and a moderately convincible m^6^A-modified predicted peak near *MAFF* (rs9610915)

To investigate the possible m^6^A methylation sites of *TOM1L1* (rs1802212; rs4829), *MAFF* (rs2267372; rs9610915), and *TNS1* (rs1424916), their transcript sequences were predicted at the SRAMP website. The results showed that five m^6^A-SNPs belonged to the high or medium confidence category, including rs1802212 (*TOM1L1*), rs4829 (*TOM1L1*), rs2267372 (*MAFF*), rs9610915 (*MAFF*), and rs1424916 (*TNS1*) (Table 1). Based on the SRAMP website, we found moderately-highly convincible m^6^A-modified predicted peaks near *TOM1L1* (rs4829) and a moderately convincible m^6^A-modified predicted peak near *MAFF* (rs9610915) (Figure 6).

**Figure 7.**
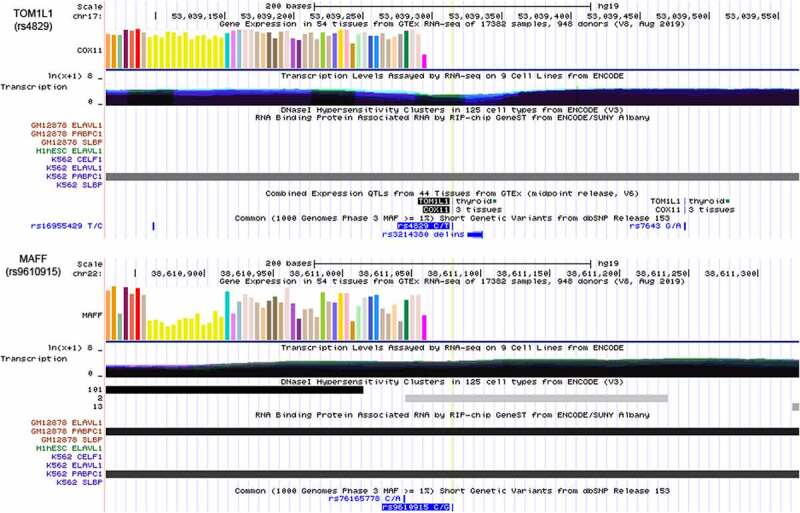
Integrative analysis of the potential function of rs4829 and rs9610915 SNPs by querying USCS. The SNP rs4829 was located on the 3’UTR of the *TOM1L1* gene on chromosome 17 and rs9610915 was located on the 3’UTR of the *MAFF* gene on chromosome 22. Further, RIP-chip GeneST from ENCODE/SUNY Albany data suggested that polyadenylate-binding protein cytoplasmic 1 (*PABPC1*) might have a potential interaction with rs4829 (*TOM1L1*) and rs9610915 (MAFF)

We next used the USCS browser to analyze the potential functions of the m^6^A-SNPs. An integrative analysis of the potential functions of rs4829 and rs9610915 is shown in Figure 7. The SNP rs4829 was located in the 3ʹUTR of *TOM1L1* on chromosome 17 and rs9610915 was located in the 3ʹUTR of *MAFF* on chromosome 22 (Figure 7). This finding suggested that these two SNPs may affect the m6A modification site and regulate gene expression to participate in breast cancer pathogenesis.

## 4 Discussion

m^6^A plays an important role in breast cancer, but the effect of m^6^A-SNPs in breast cancer is unclear. Identifying m^6^A-SNPs associated with breast cancer will provide a new annotation for the pathogenic mechanism of breast cancer risk loci identified by GWAS; therefore, we identified m^6^A-SNPs associated with breast cancer and explored their potential functions. We found 113 m^6^A-SNPs that were associated with breast cancer, and 86 m^6^A-SNPs that had eQTL signals.

Additionally, there were 31 m^6^A-SNPs belonging to the high confidence category; further, 75% of the m^6^A-SNPs were distributed in intron regions, 20% were distributed in the 3ʹUTR region, and 5% were distributed in the 5ʹUTR region. However, there were no SNPs of m^6^A regulators (*METTL3, METTL14, METTL16, WTAP, VIRMA, RBM15, FTO, ALKBH5, YTHDC1, YTHDC2, YTHDF1, YTHDF2, YTHDF3, IGF2BP1, IGF2BP2, IGF2BP3, HNRNPA2B1*, and *EIF3A*) in the m^6^A-SNPs associated with breast cancer (Table S1). This finding indicated that SNPs of these 18 m^6^A regulators may not affect breast cancer.

We also found that six corresponding genes of eight m^6^A-SNPs were differentially expressed in breast cancer, and the SNPs rs4829 and rs9610915 were located next to the m^6^A modification site in the 3ʹUTR of *TOM1L1* and *MAFF*, respectively, suggesting that these two SNPs may affect the m^6^A modification site and regulate gene expression to play a role in breast cancer. Studies have shown that *TOM1L1* is associated with breast cancer. For example, Chevalier et al. found that *TOM1L1* amplification may enhance the metastatic progression of ERBB2-positive breast cancers by regulating the *ERBB2*-driven proteolytic invasion [34,35]. There is also evidence that *MAFF* regulates cancer pathogenesis. For instance, Martínez-Hernández et al. found 17 variants of *MAFF* in chronic myeloid leukemia (CML), and the rs9610915 SNP of *MAFF* was significantly associated with CML [36]. Further, RIP-chip GeneST from the ENCODE/SUNY Albany data suggested that polyadenylate-binding protein cytoplasmic 1 (*PABPC1*) might have a potential interaction with rs4829 (*TOM1L1*) and rs9610915 (*MAFF*) (Figure 5). It is known that PABP1 participates in the initiation of translation and stabilization of mRNA and that *PABPC1* can encode PABP1 protein and promote tumor progression of gliomas and hepatocellular carcinomas [37,38]. In breast cancer, studies have indicated that *PABPC1* mediates *SNHG14*-induced oncogenic effects [39].

## 5 Conclusion

In this study, we used a comprehensive analysis of GWAS raw data and summary statistics combined with eQTL and differential gene expression analyses to identify breast cancer-associated m^6^A-SNPs. We found that two SNPs (rs4829 and rs9610915) had eQTL signals, altered gene expression, and were located next to the m^6^A modification sites in the 3ʹUTRs of their coding genes and that *PABPC1* might have a potential interaction with these two SNPs. These findings indicated that the SNPs rs4829 and rs9610915 may be potentially associated with breast cancer. However, the relationship between these m^6^A-SNPs and breast cancer risk and their potential roles in the pathogenesis of breast cancer still require clarification.

## Supplementary Material

Supplemental Material

## Highlights

1. 113 m^6^A-SNPs associated with breast cancer (5.0E-05), and 86 m^6^A-SNPs had eQTL signals.
2. SNPs rs4829 and rs9610915 had eQTL signals, altered the expression, located next to the m6A 3’UTR modification site of their coding genes.
3. SNPs rs4829 and rs9610915 may be potentially associated with breast cancer.
4. *PABPC1* might have a potential interaction with SNPs rs4829 and rs9610915.
