# Supplementary material for: Integrative genomic analysis of N6-methyladenosine-single nucleotide polymorphisms (m6A-SNPs) associated with breast cancer
Source: Bioengineered. 2021 Jun 21;12(1):2389–97. doi: 10.1080/21655979.2021.1935406 (PMC8806828; doi:10.1080/21655979.2021.1935406)
Supplement: Supplemental Material [file KBIE_A_1935406_SM5246.zip › supplementary/Table S1.docx]

Table S1. 113 m6A-SNPs associated with breast cancer passing a p value threshold of 5.0E-5.

| SNP ID | Chr | Position | m6A_ID | Gene | Gene_  region | eQTL Hit | Confidence_level | Modification_  function | Pvalue |
| --- | --- | --- | --- | --- | --- | --- | --- | --- | --- |
| rs3104793 | 16 | 52607274 | RMVar_ID_786885 | CASC16 | intron | 42 | m6A-Label-seq:(High) | Functional Loss | 2.90E-51 |
| rs12804411 | 11 | 69469432 | RMVar_ID_921856 | AP000439.3 | exon | 0 | Prediction:(Low) | Functional Gain | 1.81E-47 |
| rs9383935 | 6 | 151618713 | RMVar_ID_1369539 | CCDC170 | 3'UTR | 7 | Prediction:(Low) | Functional Loss | 3.46E-45 |
| rs11718403 | 3 | 27179940 | RMVar_ID_793899 | - | Unknown | 86 | m6A-Label-seq:(High) | Functional Loss | 2.56E-44 |
| rs4973758 | 3 | 27262218 | RMVar_ID_796997 | NEK10 | intron | 77 | m6A-Label-seq:(High) | Functional Loss | 3.09E-42 |
| rs2981428 | 10 | 121569915 | RMVar_ID_279534 | FGFR2 | intron | 1 | m6A-Label-seq:(High) | Functional Loss | 4.67E-29 |
| rs2541243 | 17 | 55013905 | RMVar_ID_595149 | STXBP4 | intron | 63 | m6A-Label-seq:(High) | Functional Loss | 3.27E-25 |
| rs1802212 | 17 | 54961293 | RMVar_ID_782932 | TOM1L1 | 3'UTR | 32 | m6A-Seal-seq:(Medium) | Functional Loss | 3.20E-19 |
| rs4573721 | 12 | 28194746 | RMVar_ID_850130 | - | Unknown | 286 | m6A-Label-seq:(High) | Functional Loss | 4.22E-18 |
| rs11049248 | 12 | 27977639 | RMVar_ID_847505 | - | Unknown | 0 | m6A-Label-seq:(High) | Functional Loss | 1.79E-17 |
| rs2666760 | 10 | 21966168 | RMVar_ID_552239 | DNAJC1 | intron | 0 | m6A-Label-seq:(High) | Functional Loss | 4.03E-15 |
| rs2807983 | 10 | 21929844 | RMVar_ID_552238 | DNAJC1 | intron | 0 | m6A-Label-seq:(High) | Functional Loss | 6.32E-15 |
| rs11917224 | 3 | 27500326 | RMVar_ID_796045 | - | Unknown | 0 | m6A-Label-seq:(High) | Functional Loss | 8.69E-15 |
| rs4149909 | 1 | 241860596 | RMVar_ID_757764 | EXO1 | CDS | 0 | miCLIP:(High) | Functional Loss | 2.93E-14 |
| rs7645905 | 3 | 4716560 | RMVar_ID_794526 | - | Unknown | 1 | m6A-Label-seq:(High) | Functional Loss | 4.29E-14 |
| rs388685 | 19 | 43914528 | RMVar_ID_763760 | ZNF45 | CDS | 16 | MeRIP-seq:(Medium) | Functional Loss | 6.1E-14 |
| rs2747655 | 6 | 152126186 | RMVar_ID_1369593 | ESR1 | 3'UTR | 3 | Prediction:(Low) | Functional Loss | 8.62E-14 |
| rs1314914 | 14 | 68236359 | RMVar_ID_981081 | RN7SL108P | exon | 0 | Prediction:(Low) | Functional Loss | 1.01E-13 |
| rs11878954 | 19 | 43780500 | RMVar_ID_764975 | KCNN4 | intron | 0 | MeRIP-seq:(Medium) | Functional Loss | 2.19E-12 |
| rs1135430 | 4 | 38787740 | RMVar_ID_761479 | - | Unknown | 14 | m6A-Label-seq:(High) | Functional Loss | 4.58E-12 |
| rs12202408 | 6 | 81583230 | RMVar_ID_721859 | TENT5A | intron | 0 | m6A-Label-seq:(High) | Functional Loss | 2.41E-11 |
| rs1063966 | 19 | 19505933 | RMVar_ID_148619 | - | Unknown | 92 | MeRIP-seq:(Medium) | Functional Loss | 3.11E-11 |
| rs1054284 | 19 | 19506144 | RMVar_ID_1113641 | GATAD2A | 3'UTR | 83 | Prediction:(Low) | Functional Gain | 7.94E-11 |
| rs6465348 | 7 | 92113288 | RMVar_ID_1415421 | AC000120.3,CYP51A1 | exon,3'UTR | 303 | Prediction:(Low) | Functional Gain | 1.11E-10 |
| rs17650901 | 17 | 45962325 | RMVar_ID_592266 | MAPT | 5'UTR | 532 | MeRIP-seq:(Medium) | Functional Loss | 1.62E-10 |
| rs76324150 | 17 | 45895867 | RMVar_ID_1068953 | MAPT-IT1,MAPT | exon,intron | 1000 | Prediction:(Low) | Functional Loss | 2.18E-10 |
| rs17574425 | 17 | 46031822 | RMVar_ID_1069086 | KANSL1 | intron | 1333 | Prediction:(Low) | Functional Gain | 2.31E-10 |
| rs2267372 | 22 | 38202227 | RMVar_ID_520365 | MAFF | exon | 35 | MeRIP-seq:(Medium) | Functional Loss | 2.49E-10 |
| rs366858 | 17 | 45649222 | RMVar_ID_1068841 | LINC02210-CRHR1,LINC02210 | intron,exon | 812 | Prediction:(Low) | Functional Loss | 3.36E-10 |
| rs385691 | 17 | 45648759 | RMVar_ID_1068836 | LINC02210-CRHR1,LINC02210 | intron,exon | 812 | Prediction:(Low) | Functional Loss | 3.63E-10 |
| rs244297 | 17 | 55164339 | RMVar_ID_1072374 | STXBP4 | 3'UTR | 63 | Prediction:(Low) | Functional Loss | 5.3E-10 |
| rs7109844 | 11 | 69415844 | RMVar_ID_921808 | AP005233.1 | exon | 0 | Prediction:(Low) | Functional Loss | 5.56E-10 |
| rs244298 | 17 | 55164026 | RMVar_ID_1072368 | STXBP4 | 3'UTR | 21 | Prediction:(Low) | Functional Loss | 6.5E-10 |
| rs6713978 | 2 | 24897982 | RMVar_ID_817849 | ADCY3 | intron | 119 | m6A-Label-seq:(High) | Functional Loss | 1.06E-09 |
| rs1017968 | 3 | 100068167 | RMVar_ID_685613 | FILIP1L | intron | 64 | m6A-Label-seq:(High) | Functional Loss | 1.24E-09 |
| rs2075744 | 11 | 1997219 | RMVar_ID_860605 | - | Unknown | 28 | MeRIP-seq:(Medium) | Functional Loss | 1.9E-09 |
| rs2075745 | 11 | 1997106 | RMVar_ID_893822 | H19 | exon | 26 | Prediction:(Low) | Functional Loss | 2.1E-09 |
| rs72885726 | 18 | 26907512 | RMVar_ID_1089518 | CHST9 | 3'UTR | 0 | Prediction:(Low) | Functional Gain | 5.67E-09 |
| rs4146689 | 10 | 21987890 | RMVar_ID_552241 | - | Unknown | 0 | m6A-Label-seq:(High) | Functional Loss | 8.16E-09 |
| rs13410999 | 2 | 24875070 | RMVar_ID_501989 | ADCY3 | intron | 39 | m6A-Label-seq:(High) | Functional Loss | 9.26E-09 |
| rs172605 | 11 | 1983880 | RMVar_ID_893791 | MRPL23-AS1 | exon | 8 | Prediction:(Low) | Functional Gain | 9.44E-09 |
| rs33949561 | 14 | 68086812 | RMVar_ID_800494 | HSALNG0102076 | intron | 0 | m6A-Label-seq:(High) | Functional Loss | 1.04E-08 |
| rs9610915 | 22 | 38215073 | RMVar_ID_778207 | MAFF | 3'UTR | 9 | MeRIP-seq:(Medium) | Functional Loss | 1.05E-08 |
| rs1509964 | 10 | 62807604 | RMVar_ID_838390 | ADO | 3'UTR | 0 | miCLIP:(High) | Functional Loss | 1.23E-08 |
| rs4824 | 1 | 155689149 | RMVar_ID_558393 | - | Unknown | 32 | MeRIP-seq:(Medium) | Functional Loss | 3.17E-08 |
| rs1424916 | 2 | 217874577 | RMVar_ID_814993 | TNS1 | intron | 4 | m6A-Label-seq:(High) | Functional Loss | 3.36E-08 |
| rs77848624 | 22 | 45878192 | RMVar_ID_1228388 | BX324167.2 | exon | 1 | Prediction:(Low) | Functional Loss | 4.49E-08 |
| rs12724120 | 1 | 121526757 | RMVar_ID_255102 | - | Unknown | 1 | m6A-Label-seq:(High) | Functional Loss | 6.46E-08 |
| rs11242115 | 5 | 132490721 | RMVar_ID_687542 | IRF1 | 5'UTR | 2 | MeRIP-seq:(Medium) | Functional Loss | 1.03E-07 |
| rs6664515 | 1 | 203873227 | RMVar_ID_1174936 | KRT8P29 | exon | 1 | Prediction:(Low) | Functional Loss | 1.15E-07 |
| rs67483387 | 17 | 45574268 | RMVar_ID_592224 | RF00017-2364,lnc-PLEKHM1-8,lnc-PLEKHM1-8:2 | intron,intron,intron | 0 | m6A-Label-seq:(High) | Functional Loss | 1.26E-07 |
| rs7430871 | 3 | 59376071 | RMVar_ID_488394 | - | Unknown | 0 | m6A-Label-seq:(High) | Functional Loss | 1.42E-07 |
| rs3835263 | 1 | 113697697 | RMVar_ID_1147568 | PHTF1 | exon | 0 | Prediction:(Low) | Functional Loss | 1.47E-07 |
| rs2974935 | 1 | 155212052 | RMVar_ID_1156287 | MTX1 | intron | 36 | Prediction:(Low) | Functional Loss | 1.52E-07 |
| rs59352002 | 1 | 155252939 | RMVar_ID_1156365 | FAM189B | intron | 45 | Prediction:(Low) | Functional Gain | 2.8E-07 |
| rs7207285 | 17 | 42404497 | RMVar_ID_1066198 | CAVIN1 | 3'UTR | 18 | Prediction:(Low) | Functional Loss | 3.98E-07 |
| rs7930569 | 11 | 771034 | RMVar_ID_862638 | - | Unknown | 7 | MeRIP-seq:(Medium) | Functional Loss | 4.4E-07 |
| rs1693685 | 10 | 121666644 | RMVar_ID_279538 | - | Unknown | 3 | m6A-Label-seq:(High) | Functional Loss | 4.74E-07 |
| rs4886615 | 15 | 74839320 | RMVar_ID_1007355 | ULK3 | exon | 10 | Prediction:(Low) | Functional Gain | 5.53E-07 |
| rs77897927 | 8 | 127228933 | RMVar_ID_319764 | CASC19,CASC19:2,CASC19:3,CASC19:4,CASC19:5 | intron,exon,exon,exon,exon | 0 | MeRIP-seq:(Medium) | Functional Loss | 6.55E-07 |
| rs11654121 | 17 | 42645443 | RMVar_ID_728748 | - | Unknown | 32 | m6A-Label-seq:(High) | Functional Loss | 6.84E-07 |
| rs4829 | 17 | 54961954 | RMVar_ID_781699 | TOM1L1 | 3'UTR | 14 | miCLIP:(High) | Functional Loss | 0.000001 |
| rs72795121 | 5 | 132302631 | RMVar_ID_823426 | lnc-P4HA2-3 | intron | 1 | MeRIP-seq:(Medium) | Functional Loss | 1.18E-06 |
| rs17514713 | 22 | 28786082 | RMVar_ID_516476 | - | Unknown | 1 | MeRIP-seq:(Medium) | Functional Loss | 1.43E-06 |
| rs73172318 | 21 | 14996258 | RMVar_ID_357526 | - | Unknown | 0 | MeRIP-seq:(Medium) | Functional Loss | 1.54E-06 |
| rs3774729 | 3 | 63996406 | RMVar_ID_1305887 | ATXN7 | CDS | 0 | Prediction:(Low) | Functional Gain | 3.58E-06 |
| rs2890975 | 9 | 13931822 | RMVar_ID_642864 | LINC00583 | intron | 0 | m6A-Label-seq:(High) | Functional Loss | 3.6E-06 |
| rs4792891 | 17 | 45896132 | RMVar_ID_1068959 | MAPT-IT1,MAPT | exon,intron | 1 | Prediction:(Low) | Functional Gain | 3.66E-06 |
| rs3219489 | 1 | 45331833 | RMVar_ID_1189779 | MUTYH,AL451136.1 | intron,CDS | 18 | Prediction:(Low) | Functional Loss | 3.67E-06 |
| rs10416177 | 19 | 43797391 | RMVar_ID_1124787 | LYPD5 | 3'UTR | 14 | Prediction:(Low) | Functional Loss | 3.9E-06 |
| rs2234458 | 11 | 65871903 | RMVar_ID_919384 | EFEMP2 | intron | 5 | Prediction:(Low) | Functional Loss | 4.68E-06 |
| rs34716807 | 10 | 117486338 | RMVar_ID_872887 | EMX2OS | intron | 0 | Prediction:(Low) | Functional Gain | 5E-06 |
| rs529224 | 1 | 100352622 | RMVar_ID_637651 | CDC14A | intron | 2 | MeRIP-seq:(Medium) | Functional Loss | 5.29E-06 |
| rs2531988 | 16 | 3978416 | RMVar_ID_335564 | ADCY9 | intron | 1 | m6A-Label-seq:(High) | Functional Loss | 5.42E-06 |
| rs6706526 | 2 | 171523880 | RMVar_ID_602757 | - | Unknown | 9 | m6A-Label-seq:(High) | Functional Loss | 5.72E-06 |
| rs1799918 | 19 | 12891586 | RMVar_ID_1108518 | GCDH | intron | 27 | Prediction:(Low) | Functional Loss | 5.84E-06 |
| rs739468 | 9 | 133461126 | RMVar_ID_715939 | lnc-SLC2A6-3 | intron | 7 | m6A-Label-seq:(High) | Functional Loss | 6.93E-06 |
| rs6456381 | 6 | 20901001 | RMVar_ID_804308 | CDKAL1 | intron | 0 | m6A-Label-seq:(High) | Functional Loss | 7.12E-06 |
| rs56909107 | 1 | 46342552 | RMVar_ID_1190200 | NSUN4 | intron | 198 | Prediction:(Low) | Functional Gain | 7.24E-06 |
| rs17357621 | 1 | 46342025 | RMVar_ID_1190198 | NSUN4 | intron | 198 | Prediction:(Low) | Functional Gain | 7.4E-06 |
| rs68083747 | 1 | 46341744 | RMVar_ID_638541 | - | Unknown | 198 | m6A-Label-seq:(High) | Functional Loss | 7.44E-06 |
| rs2582796 | 1 | 119801511 | RMVar_ID_753562 | REG4 | intron | 9 | m6A-Label-seq:(High) | Functional Loss | 7.46E-06 |
| rs2853641 | 1 | 156211979 | RMVar_ID_753748 | SLC25A44 | 3'UTR | 42 | DART-seq:(High) | Functional Loss | 7.8E-06 |
| rs3741194 | 11 | 66858763 | RMVar_ID_858079 | LRFN4 | CDS | 6 | MeRIP-seq:(Medium) | Functional Loss | 9.3E-06 |
| rs3757138 | 6 | 26375875 | RMVar_ID_809012 | BTN3A2 | 3'UTR | 73 | miCLIP:(High) | Functional Loss | 1E-05 |
| rs10447119 | 5 | 50592069 | RMVar_ID_440310 | - | Unknown | 16 | m6A-Label-seq:(High) | Functional Loss | 1.07E-05 |
| rs2073531 | 6 | 26375028 | RMVar_ID_1373098 | BTN3A2 | intron | 73 | Prediction:(Low) | Functional Loss | 1.13E-05 |
| rs2235265 | 22 | 38221326 | RMVar_ID_776128 | - | Unknown | 2 | MeRIP-seq:(Medium) | Functional Loss | 1.14E-05 |
| rs11755069 | 6 | 4414918 | RMVar_ID_179288 | lnc-C6orf201-22 | intron | 13 | m6A-Label-seq:(High) | Functional Loss | 1.18E-05 |
| rs6005881 | 22 | 28787145 | RMVar_ID_776539 | CCDC117 | 3'UTR | 45 | DART-seq:(High) | Functional Loss | 1.47E-05 |
| rs41293277 | 1 | 46340878 | RMVar_ID_1190194 | NSUN4 | CDS | 198 | Prediction:(Low) | Functional Gain | 1.48E-05 |
| rs12905221 | 15 | 50367439 | RMVar_ID_1000267 | GABPB1-AS1 | intron | 17 | Prediction:(Low) | Functional Loss | 1.57E-05 |
| rs13248350 | 8 | 22710617 | RMVar_ID_614423 | AC055854.1 | intron | 0 | m6A-Label-seq:(High) | Functional Loss | 1.71E-05 |
| rs2023472 | 6 | 30108087 | RMVar_ID_1375857 | TRIM31 | CDS | 1 | Prediction:(Low) | Functional Gain | 1.83E-05 |
| rs41308922 | 9 | 116686877 | RMVar_ID_1447774 | ASTN2 | intron | 1 | Prediction:(Low) | Functional Gain | 1.83E-05 |
| rs36081 | 5 | 149049441 | RMVar_ID_822358 | lnc-ABLIM3-3 | intron | 9 | m6A-Label-seq:(High) | Functional Loss | 1.86E-05 |
| rs200546917 | 1 | 121387910 | RMVar_ID_755936 | SRGAP2-AS1 | intron | 0 | MeRIP-seq:(Medium) | Functional Loss | 2.16E-05 |
| rs615767 | 18 | 69221582 | RMVar_ID_566677 | - | Unknown | 0 | m6A-Label-seq:(High) | Functional Loss | 2.27E-05 |
| rs3811436 | 1 | 45660208 | RMVar_ID_638299 | GPBP1L1 | 5'UTR | 170 | m6A-REF-seq:(High) | Functional Loss | 2.44E-05 |
| rs4649207 | 1 | 24210252 | RMVar_ID_1165017 | LINC02800 | exon | 23 | Prediction:(Low) | Functional Gain | 2.53E-05 |
| rs3881547 | 10 | 38453934 | RMVar_ID_839227 | AL133216.1 | intron | 19 | m6A-Label-seq:(High) | Functional Loss | 2.58E-05 |
| rs6962151 | 7 | 100430861 | RMVar_ID_829742 | - | Unknown | 63 | MeRIP-seq:(Medium) | Functional Loss | 2.61E-05 |
| rs10471798 | 5 | 50368927 | RMVar_ID_821787 | - | Unknown | 81 | m6A-Label-seq:(High) | Functional Loss | 2.61E-05 |
| rs13247705 | 7 | 102448711 | RMVar_ID_1419024 | ORAI2 | 3'UTR | 8 | Prediction:(Low) | Functional Gain | 3E-05 |
| rs8188046 | 5 | 50194828 | RMVar_ID_440238 | - | Unknown | 1 | MeRIP-seq:(Medium) | Functional Loss | 3.03E-05 |
| rs1363580 | 12 | 68669088 | RMVar_ID_952794 | RAP1B | 3'UTR | 8 | Prediction:(Low) | Functional Loss | 3.61E-05 |
| rs9971342 | 10 | 21590818 | RMVar_ID_552139 | - | Unknown | 0 | m6A-Label-seq:(High) | Functional Loss | 3.66E-05 |
| rs7792525 | 7 | 100374499 | RMVar_ID_829541 | PILRA | intron | 102 | MeRIP-seq:(Medium) | Functional Loss | 3.87E-05 |
| rs1040 | 6 | 169216252 | RMVar_ID_805449 | THBS2 | 3'UTR | 8 | MeRIP-seq:(Medium) | Functional Loss | 3.93E-05 |
| rs1780138 | 10 | 38212726 | RMVar_ID_839673 | AL117339.4 | intron | 62 | m6A-Label-seq:(High) | Functional Loss | 4.44E-05 |
| rs34667451 | 19 | 19506208 | RMVar_ID_148627 | - | Unknown | 1 | MeRIP-seq:(Medium) | Functional Loss | 4.54E-05 |
| rs79703513 | 15 | 75464727 | RMVar_ID_1007891 | PTPN9 | 3'UTR | 41 | Prediction:(Low) | Functional Loss | 4.6E-05 |
| rs76550488 | 15 | 75464709 | RMVar_ID_1007889 | PTPN9 | 3'UTR | 41 | Prediction:(Low) | Functional Loss | 4.62E-05 |
